# Preparation and Physical Properties of Red Mud Based Artificial Lightweight Aggregates

**DOI:** 10.3390/ma18163741

**Published:** 2025-08-10

**Authors:** Rubin Han, Yunrui Zhao, Hui Luo, Hongxiu Leng, Wenbo Wu, Bukai Song, Bao-Jie He

**Affiliations:** 1 School of Civil and Ocean Engineering, Jiangsu Ocean University, Lianyungang 222005, China; 19816221775@163.com (R.H.); zhaoyr997997@163.com (Y.Z.); leng010124@163.com (H.L.); wuwenbol@163.com (W.W.); 2024220518@jou.edu.cn (B.S.); 2 Institute of Oceanology, Chinese Academy of Sciences, Qingdao 266071, China; 3 Faculty of Built Environment, University of New South Wales, Sydney, NSW 2052, Australia; 4 Centre for Climate-Resilient and Low-Carbon Cities, School of Architecture and Urban Planning, Chongqing University, Chongqing 400045, China

**Keywords:** red mud, fly ash, physical characteristics, lightweight aggregates, heavy metals

## Abstract

Highly alkaline and highly toxic red mud and other bulk industrial solid wastes become severely accumulated, posing huge risks such as soil degradation and environmental pollution. It is urgent to develop a long-term and stable resource disposal method. In the present research, artificial lightweight aggregates were fabricated utilizing industrial solid residues including red mud, phosphate tailing powder, and fly ash as raw materials. The physical characteristics, microstructure, heavy metal leaching attributes, and freeze–thaw resistance under different mixed water and curing conditions were studied. The results showed that, under the optimal curing condition (steam curing temperature of 80 °C and curing time of 10 h), lightweight aggregates exhibited the best comprehensive performance, with favorable trends in bulk density, apparent density, softening coefficient, and 1 h water absorption. In addition, the impact of extending the curing time on the further enhancement of the cylinder crush strength is limited. The microscopic morphology study showed that the hydration products in lightweight aggregates are primarily N-A-S-H and C-(A)-S-H, forming a strong colloidal structure and evenly dispersed on the particle surface, thereby improving its strength. Moreover, the heavy metal leachates (Cr, Pb, As, Cu, and Ni) from the lightweight aggregates met the environmental discharge criteria for non-hazardous substances.

## 1. Introduction

Red mud (RM) is a type of industrial solid waste produced in the Al smelting industry when Al_2_O_3_ is extracted [1,2], which exhibits the features of high alkalinity, a high heavy metal content, complex composition, and small particle size [3,4,5]. According to statistics, the global production of RM is predominantly distributed in several regions. China accounts for 28.2%, North and South America together hold 33.4%, Oceania makes up 22.4%, and Europe constitutes 12.9%. The production of RM is increasing at a rate of 150 million tons annually, with an inventory of about 3 billion tons. However, its resource utilization rate remains below 10% [6,7], a challenge echoed in international research highlighting RM as a pressing issue for circular economy transition across continents [2,8,9]. From a regulatory perspective, this urgency is underscored by global initiatives: for instance, the European Union’s Circular Economy Action Plan emphasizes hazardous waste valorization [10,11], while China’s “14th Five-Year Guidance on Effective Utilization of Bulk Solid Wastes” and “14th Five-Year Circular Economy Development Plan” (2021) specifically target RM, mandating enhanced resource treatment of hazardous solid wastes and aiming for a 79% bulk solid waste utilization rate by 2025 [12]. Against this backdrop, exploring sustainable RM reuse pathways such as RM-based artificial lightweight aggregate development aligns with both international research priorities and global regulatory frameworks, addressing the critical need to transform RM from waste to resource, so it is a central focus of this study within the broader environmental engineering context [13].

At present, the main disposal methods of RM in China include deep-pit landfill, dam stacking, and direct open-pit stacking [14,15]. However, heavy metals and alkaline substances will gradually penetrate into the ground during the long-term storage of RM, resulting in the pollution of groundwater resources and land resources [16,17]. At the same time, RM will produce dust after water loss, causing pollution of the air. Especially, the method of constructing and storing RM in dams consumes a vast quantity of land resources as well as carries the risk of dam failure. In severe situations, this can present a significant hazard to the natural setting and human safety [15]. Consequently, it is essential to seek a safer and more efficient approach for treating RM to achieve the transformation from waste to value-added materials [18].

In the past few years, the preparation of artificial lightweight aggregate by solid waste has gained the attention of many scholars. The preparation methods of artificial lightweight aggregate include sintering and non-sintering. With sintering preparation methods, Molineux et al. [19] manufactured lightweight aggregates using RM and pulverized coal fuel ash. They discovered that, when the RM content was 44% and the sintering temperature was 1200 °C, the lightweight aggregates achieved their maximum compressive strength. However, these aggregates exhibited a high absorption rate of water. Sun et al. [20] utilized RM and municipal solid waste incineration bottom ash to prepare lightweight aggregates. The results suggested that, under a sintering temperature equal to 1070 °C when the optimal ratio of RM and incineration bottom ash is 1:1, the lightweight aggregate has a 0.8% water absorption rate and 27.11 MPa particle strength. Although the sintering preparation method can obtain better mechanical properties, it still has the disadvantages of high expense, enhanced energy usage, and reduced thermal insulation efficiency [21].

Based on this, researchers have sought low-cost and environmentally friendly methods to prepare unburned lightweight aggregates. Shi et al. [4] engaged in the preparation of lightweight aggregates using fly ash (RCF), carbide slag, and RM, and found that, for the best curing, it should stand for 24 h first and then be subjected to 12 h steam curing at a temperature equal to 80 °C. The findings demonstrated that the apparent density (*ρ*_ap_) of the lightweight aggregate was 1400 kg/m^3^, the bulk density (*ρ*_bu_) was 770 kg/m^3^, and the softening coefficient was 0.95. Zhao et al. [22] utilized industrial solid waste and carbide slag for producing lightweight aggregates, and then examined how mixed water and curing approaches affect the physical traits of lightweight aggregates. The results show that, when the steam curing is carried out at 60 °C for 12 h, the optimum cylindrical compressive strength of the lightweight aggregate is 8.7 MPa, which exceeds that of natural curing by 180.65%. The reason is that the enhanced temperature promotes the hydration reaction, consequently producing C-(A)-S-H gel and hydrotalcite. Colangelo et al. [23] prepared non-sintering lightweight aggregate with a cylinder compressive strength of 0.5–8.0 MPa, a *ρ*_ap_ value equal to 1700–1900 kg/m^3^, and water absorption equal to 3–24% by employing cement kiln ash, blast furnace slag powder, and marble waste. These research findings demonstrate that non-sintering artificial lightweight aggregates are an eco-friendly and highly efficient building material, offering minimal energy usage, cost-effectiveness, and excellent mechanical properties, along with lightweight characteristics. However, only a few researchers [24,25,26,27,28,29,30] have studied the environmental pollution brought about during the preparation of lightweight aggregates with industrial solid wastes. Hence, the feasibility of using RM in combination with other solid waste to manufacture lightweight aggregates under various steam curing and time conditions should be further examined.

Herein, RM is used as the raw material. In coordination with solid wastes from multiple sources (phosphate tailings (PT), fly ash (FA), and mineral powder (MP)), artificial lightweight aggregates made of RM are prepared. The aim is to investigate how different mixed water contents and curing conditions influence the physical characteristics (softening coefficient, water absorption, void ratio (v), *ρ*_bu_, cylinder crush strength, and *ρ*_ap_), heavy metal leaching attributes, and freeze–thaw resistance of RM-LWA. Furthermore, the mineral composition and microstructure of both raw materials and artificial lightweight aggregates derived from RM were analyzed using a range of characterization techniques. Specifically, XRF was employed to determine elemental composition, FTIR for identifying functional groups, XRD to analyze crystalline phases, EDS for microzone chemical composition analysis, and SEM to observe microstructural morphology. This study innovatively guides the synergistic use of different solid wastes when manufacturing artificial lightweight aggregates through the innovative methods of RM, PT, FA, and MP.

## 2. Materials and Method

### 2.1. Raw Materials

**Figure 1:**
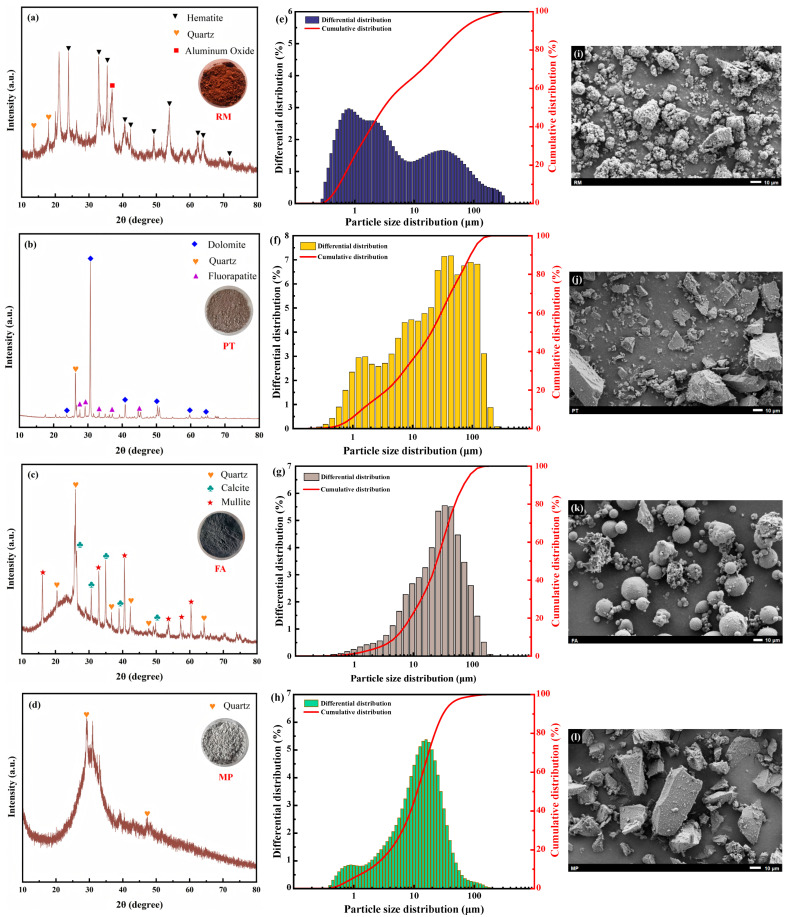
The XRD patterns, particle size distribution, and SEM images of the raw materials are as follows: For RM, they are presented in (**a**,**e**,**i**); for PT, in (**b**,**f**,**j**); for FA, in (**c**,**g**,**k**); and for MP, in (**d**,**h**,**l**).

The primary raw materials employed in this work are RM, PT, FA, and MP. As shown in Figure 1, the microstructure, mineral composition, and particle size distribution of the raw materials are presented in specific subfigures, with characterization performed using SEM, XRD, and a laser-based particle size analyzer. Specifically, the results for RM are shown in Figure 1a,e,i; those for PT in Figure 1b,f,j; FA in Figure 1c,g,k; and MP in Figure 1d,h,l.

The elemental distribution of the raw materials was explored by employing XRF. It is evident from Table 1 that these are the primary chemical components of the raw materials. It was revealed by the results that RM contained 4.46% of Na_2_O, indicating that the alkalinity was high. The content of Fe_2_O_3_ is 50.10%, showing slight magnetism. Furthermore, the SiO_2_ contents of PT and FA are as high as 35.12% and 37.16%, respectively, while the CaO content in MP is 44.05%, which has potential activity. The LOI^a^ parameters show that the ignition losses of RM and PT are 9.16% and 7.26%, respectively, while the ignition loss of FA is 1.43%.

### 2.2. Preparation of RM-LWA

**Figure 2:**
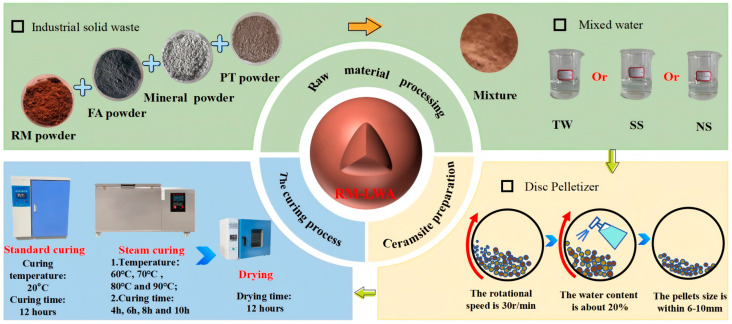
RM-LWA manufacturing steps: raw material processing, followed by the preparation and the curing processes.

As shown in Figure 2, the production process for artificial lightweight aggregates is depicted. Building upon prior research findings, the suitable ranges of the calcium–silicon ratio (Ca/Si) and calcium–aluminum ratio (Ca/Al) of raw materials for preparing unburned lightweight aggregates are determined to be 0.68–1.15 and 1.12–1.22, respectively [4,12]. In this study, a fixed mass ratio of RM:PT:FA:MP = 3:3:3:1 was adopted, and calculations showed that the mixed raw materials under this ratio had a Ca/Si of 0.71 and a Ca/Al of 1.18, both conforming to the required composition ranges, thus validating its use for preparation.

Since PT cannot be directly used, it was first dried in an oven at 100 °C for 24 h until completely dry, then crushed into powder. Thereafter, the four raw materials were accurately weighed in accordance with the specified proportion, poured into a mixer, and stirred for more than 30 min until thoroughly mixed. Subsequently, the blended raw materials were transferred to a disc granulator running at 30 r/min for pelletization, during which tap water (TW), sodium silicate solution (SS, modulus = 1.0), and sodium hydroxide–sodium silicate solution (NS) were evenly sprayed onto the mixture, each accounting for 20% of the dry weight. After up to 20 min of granulation, spherical aggregates with a diameter range of 6–10 mm were produced, and the pellet formation procedure was carried out in accordance with China’s national standard GB/T 17431.1-2010 [31].

### 2.3. Curing Conditions

The lightweight aggregates sourced from RM were kept in a 20 ± 2 °C setting for a duration of 24 h. The specific curing methods are given in Table 2. Samples A1–A3 underwent the curing process within a standard SHBY-40B curing box that maintained stable temperature and moisture levels for 12 h. The remaining samples were subjected to steam curing.

Samples B1–B3, C1–C3, D1–D3, and E1–E3 were cured at 60 °C, 70 °C, 80 °C, and 90 °C, respectively, with all curing durations set at 12 h. Regarding curing duration, samples F1–F3, G1–G3, H1–H3, and I1–I3 were cured at a constant temperature of 80 °C for 4 h, 6 h, 8 h, and 10 h, respectively. The samples were then placed in an electrothermal blast drying oven (DHG9101) for 12 h, and ultimately RM-LWA was prepared. This was carried out to investigate the impact of the steam curing temperature and curing time on the performance of RM-LWA. The whole preparation process conforms to the standard Lightweight Aggregates and Their Test Methods (GB/T 17431.1-2010) [31].

### 2.4. Testing Techniques

In this study, the testing of performance indices such as cylinder compressive strength, density, and water absorption of RM-LWA was conducted in strict accordance with the Chinese standard Lightweight Aggregates and Their Test Methods—Part 2: Test Methods for Lightweight Aggregates (GB/T 17431.2-2010) [32]. Meanwhile, to further optimize the operability and stability of the testing process, on the basis of the aforementioned standard framework, this study reasonably drew on the mature operational procedures developed in previous performance testing of similar materials by our research group [12], laying a solid foundation for the reliability of the research data.

#### 2.4.1. Determination of Cylinder Crush Strength

First, 5 L samples with grains nominally sized between 5 and 10 mm were crushed, and the sample was loaded with a pressure-bearing cylinder with the bottom of the cylinder to the upper mouth of the cylinder. For 3 s, the material was positioned on the concrete test vibration table, and then, loaded at the cylinder’s upper opening, the material underwent 5-second vibration on the shaker. Subsequently, the cylinder’s end was either scraped or filled. The pressure cylinder centered on the lower plate of the press was uniformly loaded at a speed of 300–500 N per second. The pressure value was recorded when the pressing depth of the stamping die reached 20 mm. The calculation formula for cylinder crush strength is given below:

(1) $$f_{a}=\frac{p_{1}+p_{2}}{F}$$

Among them, *f_a_* represents the cylinder compressive strength (MPa), *p*_1_ indicates the pressure value (N) when the indentation depth denotes 20 mm, *p*_2_ signifies the quality of the stamping die (N), whereas *F* indicates the pressure-bearing area (*F* = 10,000 mm^2^).

#### 2.4.2. Determination of 1 h Water Absorption

The dry weights of multiple artificial lightweight aggregates were measured. Next, all the artificial lightweight aggregates were submerged in a water-filled beaker (if any artificial lightweight aggregates were floating, a handled circular metal disc was used to submerge them). After an hour of standing, they were removed and filtered through a sieve of 2.36 mm mesh for approximately 60–120 s. The artificial lightweight aggregate was poured onto dried towel, and the towel was grasped at two ends to form a groove shape. Following a process of moving the artificial lightweight aggregate across the towel to ensure proper saturation, when in a surface-saturated and dry state, the mass *m*_1_ of the aggregates was determined. The three measured values were averaged to obtain the test result. The formula for calculating the 1 h water absorption is:

(2) $$\omega_{a}=\frac{m_{1}-m_{0}}{m_{0}}$$

Among them, *ω_a_* is the water absorption of lightweight aggregates for 1 h (%), *m*_0_ is the dry weight of lightweight aggregates (g), *m*_1_ indicates the mass of lightweight aggregates in a saturated-surface dry condition (g).

#### 2.4.3. Determination of Softening Coefficient

First, 10 L of specimens having a nominal particle size range of 5–10 mm underwent sieving. Five liters of these were portioned into three equal amounts. Each portion was weighed and then submerged in water. The specimens were submerged in water for one hour. Subsequently, they were eliminated to create samples with a specific moisture state suitable for the subsequent test requirements. In the experiment, the crush strength of cylinders was measured separately for dry samples and for samples in a state where the surface was saturated while the interior remained dry. The formula used to calculate the softening coefficient is as follows:

(3) $$\Psi =\frac{f_{1}}{f_{0}}$$

Among them, *Ψ* denotes the softening coefficient of artificial lightweight aggregates. The cylindrical crushing strength (in MPa) of these aggregates under dry conditions is represented by *f*_0_. In contrast, *f*_1_ denotes the cylindrical compressive strength (in MPa) of dry artificial lightweight aggregates following a 1 h immersion in water, during which their surface reaches a saturated state.

#### 2.4.4. Determination of Bulk and Apparent Density

(1) Apparent density

After the water absorption test was completed, the value of *ρ*_ap_ was measured further by the drainage method. After filtering, the surface moisture of the artificial lightweight aggregates was removed via a damp cloth and placed in a measuring cylinder containing a certain amount of water. The increase in the liquid level was then measured. The two measured values were averaged to yield the experimental result. The value of *ρ_ap_* was calculated using:

(4) $$\rho_{ap}=\frac{m_{0}\times 1000}{\Delta V}$$

where *m*_0_ represents the dry mass of artificial lightweight aggregates (in grams), while ∆*V* refers to the liquid level rise in the measuring cylinder before and after the aggregates are placed into it (in milliliters).

(2) Bulk density

The so-called loose bulk density, which is the bulk density of artificial lightweight aggregates, is an important parameter for assessing the compactness grade of the lightweight aggregates made artificially. A sampling spoon was used to evenly pour the artificial lightweight aggregates that were dried to constant weight from a position 50 mm above the opening of the container, and the artificial lightweight aggregates were allowed to descend naturally, ensuring it did not strike the measuring cylinder. It was ensured that the artificial lightweight aggregates corresponded to the upper edge of the volumetric measure as seen from a horizontal perspective and the artificial lightweight aggregates with smaller particle sizes filled the surface depressions. The mass of the artificial lightweight aggregates in the volumetric measure was measured at this time. The value of *ρ_bu_* (kg/m^3^) was calculated using the following formula:

(5) $$\rho_{bu}=\frac{\left(m_{t}-m_{v}\right)\times 1000}{V}$$

where *m_t_* indicates the combined mass of the lightweight aggregates and the measuring cylinder (kg), *m_v_* refers to the mass of the measuring cylinder alone (kg), and *V* stands for the volume of the measuring cylinder (L).

#### 2.4.5. Determination of Void Ratio

Using the values of *ρ_ap_* and *ρ_bu_* obtained for the sample, the value of v between particles in the natural state of accumulation is worked out, and it can be computed from the equation below:

(6) $$\nu =\left(1-\frac{\rho_{bu}}{\rho_{ap}}\right)\times 100$$

where *ν* indicates the porosity.

#### 2.4.6. Microstructural Analysis

This study utilized a scanning electron microscope (SEM, JSM-7200, JEOL Ltd., Tokyo, Japan) to detect the microstructure. EDS characterization was employed to analyze the distribution of elements in lightweight aggregate materials. For X-ray diffractometer analysis (XRD), a Rigaku ultima4 polycrystalline powder diffractometer (Tokyo, Japan) with a Cu target was utilized, and it was operated at a current equal to 40 mA, an incident wavelength λ of 0.154 nm, and a voltage equaling 40 kV. These parameters served as the basis for identifying the phase composition of artificial lightweight aggregates under different temperature conditions. A Thermo Scientific Nicolet iS50 FTIR spectrometer (Waltham, MA, USA) was employed for the FTIR test. The resolution of this spectrometer was 2 cm^−1^, and the wavenumber spanned from 400 to 4000 cm^−1^.

#### 2.4.7. Freeze-Thaw Cycling Analysis

The freeze–thaw cycle test method was formulated in accordance with the “Standard for Test Methods of Long-term Performance and Durability of Ordinary Concrete” (GB/T 50082-2009) [33]. Initially, a 2 kg portion of the specimen was immersed in water for two hours to enable the aggregates to attain the water absorption saturation state. Next, the specimen was removed, its surface was wiped dry, and it was weighed. After weighing, the samples were placed in a concrete rapid freeze–thaw tester to carry out the freeze–thaw test. For the freeze–thaw device, the temperatures for freezing and thawing processes were adjusted to −16 °C (freezing stage) and 25 °C (thawing stage). The cold–heat transition duration was 600 s and the total count of freeze–thaw cycles was set at 20. Following each cycle, the test pieces were removed, the moisture on their surfaces was wiped off until dry, and they were weighed to calculate the mass loss rate.

(7) $$\Delta W_{ni}=\frac{W_{0i}-W_{ni}}{W_{0i}}\times 100$$

where ∆*W_ni_* denotes the percentage of mass lost by the *i*-th specimen following N freeze–thaw cycles. *W*_0*i*_ represents the mass (in grams) of the *i*-th specimen before the freeze–thaw cycling test, whereas *W_ni_* signifies the mass (in grams) of the *i*-th specimen after N freeze–thaw cycles.

#### 2.4.8. Leaching Properties of Heavy Metals

The heavy metal leachability was analyzed via inductively coupled plasma optical emission spectrometry (ICP-OES) in accordance with HJ557-2010 [34], namely “Solid Waste-Extraction Procedure for Leaching Toxicity-Horizontal Vibration Method” in the present research. Following 28 days of curing, 100 g of dried, crushed aggregates (smaller than 3 mm) were put into a glass jar, with deionized water added at a liquid-to-solid ratio of 10:1 (L/kg). The bottle was oscillated on a reciprocating shaker (110 cycles/min, 40 mm amplitude) for 8 h, followed by 16 h of standing. The leaching solution was then extracted for heavy metal analysis.

## 3. Results and Discussion

### 3.1. Physical Properties of RM-LWA

#### 3.1.1. Cylinder Crush Strength

**Figure 3:**
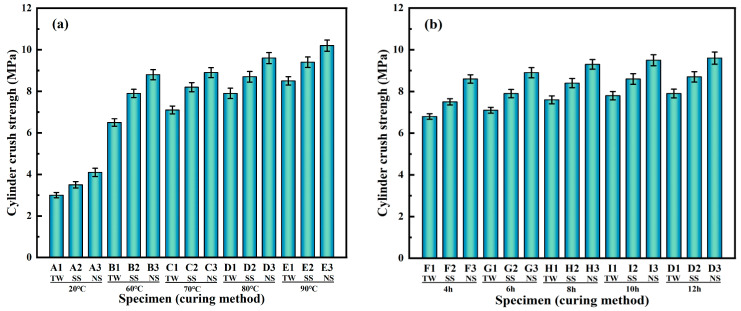
Cylinder crush strength vs. temperature (**a**) and time (**b**).

Figure 3a illustrates the impacts of various mixed water (TW, SS, and NS) and curing temperatures (20, 60, 70, 80, and 90 °C) on the cylindrical crushing strength of RM-LWA. At 20 °C, the findings indicated that the cylindrical crushing strength of red-mud-based lightweight aggregates produced using SS and NS is respectively 16.7% and 33.3% greater in comparison to that of artificial lightweight aggregates made with TW. The reason is that the high alkalinity of NS boosts the excitation effect, suggesting that, for the formation of artificial lightweight aggregates, NS is far more effective than SS. Moreover, the cylindrical crushing strength of RM-LWA obtained through steam curing was significantly greater than that of specimens produced under standard curing regimes. The cylindrical compressive strength of lightweight aggregates manufactured using three different kinds of mixing water rose as the steam curing temperature went up. Upon the curing temperature ascending from an initial 60 to 80 °C, the cylindrical crushing strength of RM-LWA produced using various kinds of mixing water experiences a notable enhancement. However, upon the steam curing temperature being elevated from 80 to 90 °C, the gains in cylindrical crushing strength are only 7.6%, 8.0%, and 6.3%, respectively, showing a slight increase. Specifically, the optimum curing temperature for RM-LWA is 80 °C. At this temperature, the cylinder crush strength of RM-LWA reaches over 90% of the strength measured at 90 °C, indicating that further increasing the temperature beyond 80 °C has a limited impact on strength enhancement. This phenomenon corresponds to the findings of Shi et al. [4]. Furthermore, RM-PT activated FA-MP more quickly when the curing temperature was increased compared to natural curing. Consequently, the decomposition and polymerization reactions occur at a swifter pace, resulting in the generation of a greater quantity of hydration products, making the material more compact, diminishing the internal voidage, and significantly enhancing the strength of artificial lightweight aggregates [35,36,37,38].

Figure 3b displays how the cylinder crush strength is influenced by different steam curing durations (4, 6, 8, 10, and 12 h) at a steam curing temperature of 80 °C. As the duration of steam curing is gradually extended from 4 h to 12 h, the cylinder crush strengths of the three distinct types of RM-LWA attain values of 7.9 MPa, 8.7 MPa, and 9.6 MPa, respectively. Notably, for the increase in curing time from 10 to 12 h, the cylinder crush strengths of the three types of RM-LWA rise only by 1.3%, 1.2%, and 1.1%. This indicated that prolonging the curing time barely contributed to enhancing the cylinder crush strength further. In addition, considering the possible loss during the curing process, RM-LWA’s ideal steam curing duration is 10 h.

#### 3.1.2. Softening Coefficient and 1 h Water Absorption

**Figure 4:**
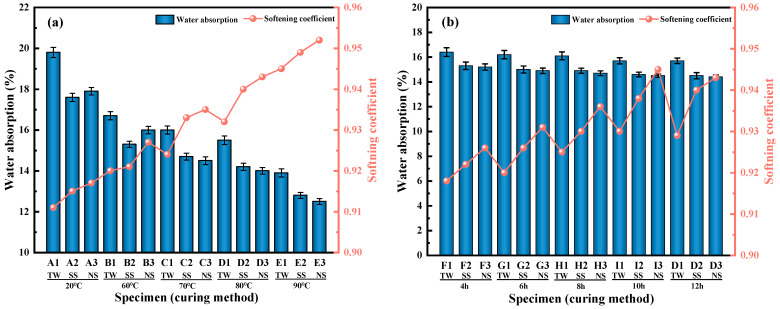
Water absorption and softening coefficient of RM-LWA: (**a**) impact of steam temperature, (**b**) impact of steam time.

The softening coefficient and 1 h water absorption of RM-LWA are influenced by various kinds of mixed water, steam curing temperature, and steam curing time, as depicted in Figure 4a,b. Figure 4a illustrates that, during the standard curing process, the 1 h water absorption rates of red-mud-based artificial lightweight aggregates fabricated using diverse mixing waters (TW, SS, and NS) are 19.8%, 17.6%, and 17.9%, respectively, and the softening coefficient varies from 0.91 to 0.92. Compared with RM-LWA manufactured by the TW process, the RM-LWA produced using a mixed water system of SS and NS exhibits a higher softening coefficient and lower water absorption. Among them, the artificial lightweight aggregate made by adding NS is superior to that made with SS. This may be because NS better stimulates the activity of the RM-LWA mixture, the number of hydrated products increases [39], the internal pores are filled, and the defects are less, so that its durability is better. In addition, as the temperature advanced slowly and steadily from 20 °C, the water absorption of RM-LWA also decreased significantly, and its softening coefficient was higher than 0.92. At a steam curing temperature of 90 °C, a water absorption rate of the RM-LWA made with NS hits the lowest value (12.5%), and its softening coefficient is as high as 0.95. This behavior can be explained by the notion that more gel structures are formed within the artificial lightweight aggregate as the temperature rises. This development reduces the interparticle connection and the presence of detrimental pores [36]. Subsequently, the infiltration of water is obstructed, and the waterproofing of RM-LWA is fortified [12,40,41].

As depicted in Figure 4b, by enhancing steam curing time, the water absorption of RM-LWA experiences only marginal fluctuations. This observation implies that the steam curing time exerts a rather negligible influence on the water absorption of the artificial lightweight aggregate. The softening coefficient of RM-LWA is also 0.91–0.95, and at temperatures equal to 80 °C and 90 °C, the softening coefficient of RM-LWA prepared by adding NS as mixed water is basically the highest, showing good water resistance and stability, which meets the Chinese standard GB/T 17431.1-2010.

#### 3.1.3. Apparent Density, Bulk Density, and Void Ratio

**Figure 5:**
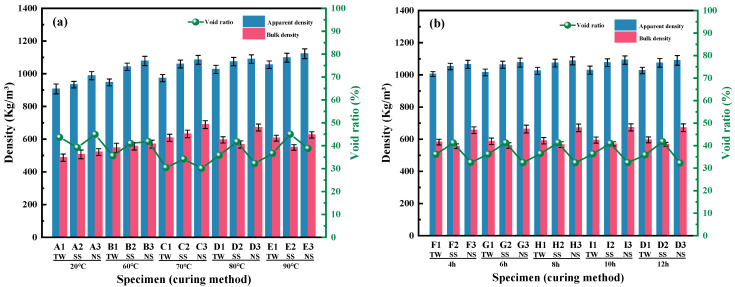
The obtained values of *ρ*_ap_, *ρ*_bu_, and v for RM-LWA: (**a**) impact of steam temperature, (**b**) impact of steam time.

Figure 5a demonstrates the changes in the values of *ρ*_ap_, *ρ*_bu_, and v for RM-LWA fabricated using various mixed water categories (TW, SS, and NS) under standard curing conditions and different steam curing temperatures. It can be found that, when the standard curing is changed into steam curing, the values of *ρ*_ap_ and *ρ*_bu_ for RM-LWA increased with an increase in temperature. The values of *ρ*_ap_ for RM-LWA fabricated with SS, TW, and NS underwent increments of 5.33%, 11.37%, and 4.09%, respectively, when the temperature reached 90 °C. Among them, the peak values of *ρ*_ap_, *ρ*_bu_, and v for RM-LWA prepared with NS were 1122.69 kg/m^3^, 626.43 kg/m^3^, and 38.75%, respectively. The high alkalinity of NS and high-temperature curing might jointly account for this phenomenon. These factors synergistically promote the hydration reaction, generating more gel products [4,42]. Consequently, the internal compactness of the material and the density of RM-LWA were enhanced as the value of v was decreased [35]. Moreover, the value of *ρ*_ap_, which varies between 907 and 1122 kg/m^3^, and the value of *ρ*_bu_, which was in the range of 487 to 689 kg/m^3^, were respectively less than 2000 kg/m^3^ and 1200 kg/m^3^ [27,43].

As can be discerned from Figure 5b, the values of *ρ*_ap_ and *ρ*_bu_ for RM-LWA exhibited a slightly upward trend when the steam curing time was increased. This minor change may be ascribed to the fact that the extended curing time facilitates the hydration reaction. The hydration products serve to fill the internal pores, thereby leading to a more compact structure [4]. Additionally, the values of *ρ*_ap_ and *ρ*_bu_ both conformed with the density requirements for lightweight artificial aggregates.

Moreover, in earlier investigations, a number of researchers have achieved the fabrication of sintered lightweight aggregates, and related studies are recapitulated in Table 3. To give an example, Qin et al. [44] produced sintered artificial lightweight aggregates with lime mud as mainly raw materials. The study demonstrated that the obtained lightweight aggregate had an apparent density of 740 kg/m^3^, a water absorption rate of 39.03%, and a cylindrical crushing strength of 4.73 MPa at a sintering temperature of 1050 °C. Jiang et al. [45] fabricated sintered lightweight aggregates by utilizing engineering excavated soil. The test results showed that, when the sintering temperature was in the range of 1100 °C to 1175 °C, the cylinder compressive strength of the lightweight aggregates could reach 5.02 MPa, demonstrating excellent performance.

In contrast to conventional high-temperature sintering methods, the current work produced unfired RM-LWA using solid wastes like RM as raw materials, incorporating an alkali activator and curing them under 80 °C steam conditions for 10 h; these aggregates demonstrated superior performance. Specifically, the cylindrical compressive strength attains 9.5 MPa, with water absorption at 14.5% and an apparent density of 1091 kg/m^3^. This approach satisfies China’s lightweight aggregate standard GB/T 17431.1-2010.

### 3.2. Microstructure Analysis

#### 3.2.1. SEM-EDS

**Figure 6:**
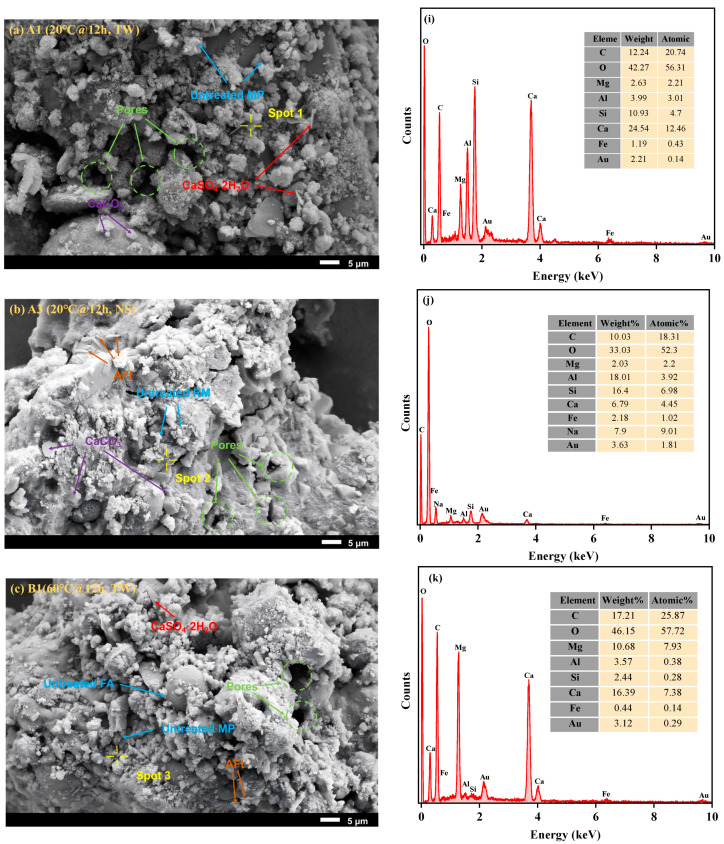

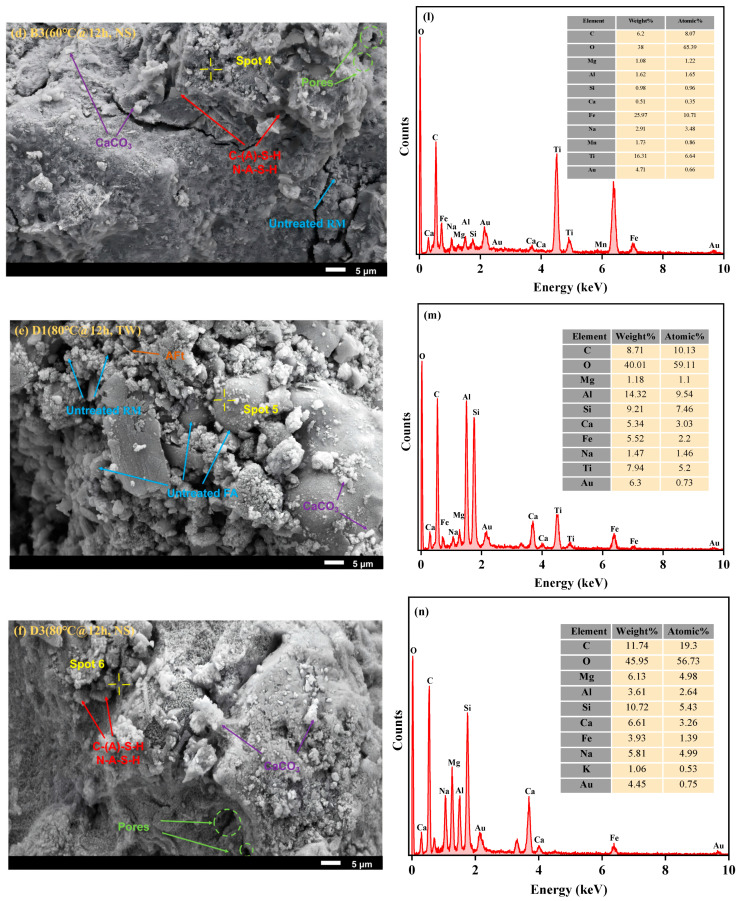

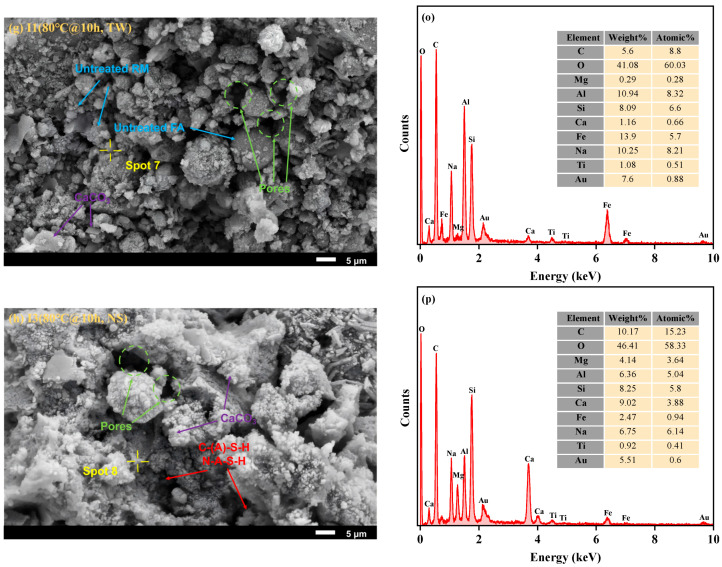
SEM images (**a**–**h**) and EDS results (**i**–**p**) of RM-LWA.

The morphologies of eight groups of samples, namely A1 (TW@20 °C, 12 h), A3 (NS@20 °C, 12 h), B1 (TW@60 °C, 12 h), B3 (NS@60 °C, 12 h), D1 (TW@80 °C, 12 h), D3 (NS@80 °C, 12 h), I1 (TW@80 °C, 10 h), and I3 (NS@80 °C, 10 h), were ascertained through SEM-EDS. The aim was to explore more thoroughly how various curing conditions affect the microporous makeup of the artificial lightweight aggregate. Figure 6 depicts that, when TW was utilized as the mixing water, the microstructures of samples A1 and B1 were relatively loose and contained numerous unreacted particles. The formation of gel products was not detected. When NS was used as the mixing liquid, a significant decrease in voids and the number of unhydrated particles was detected in sample A3. This phenomenon suggests that the potent alkalinity of NS facilitated the polymerization reaction of reactive SiO_2_ and Al_2_O_3_ present in RM and FA, thereby expediting the formation of gel products [42,51]. As the temperature rises, it becomes distinctly apparent that a greater quantity of hydration products was formed in D3 and I3. These products serve to further fill and diminish the voids among the raw material particles, gradually rendering the internal pore structure more compact. In particular, the microstructure of sample D3 showcases a remarkably outstanding performance. Perhaps this is because, by the 12-hour point for sample D3, the water curing process advances further. As a result, an increasing amount of hydration products are generated, which fill more voids and form a relatively compact colloidal network structure. Furthermore, the principal hydration products within RM-LWA are N-A-S-H and C-(A)-S-H. Throughout the hydration process, a highly robust colloidal structure is engendered, distributing homogeneously over the particle surfaces. This not only provides additional evidence for the superiority of preparing RM-LWA through the addition of the mixed water NS but also corroborates the XRD and FTIR results.

It is worth noting that the SEM-EDS results reveal some Si in the C-S-H gel is replaced by Al, which in turn further promotes the formation of C-A-S-H gel [52]. Furthermore, within samples A3, B3, D3, and I3, the Na element present in the mixed water NS has substituted the Ca element. Subsequently, it engages in a reaction with the C-A-S-H gel, giving rise to a more intricately dense 3D network of N-A-S-H gel. These gel products fill the interstitial voids between the particles, culminating in the formation of an even more compact structure. Thus, this contributes to a notable enhancement in strength [53].

#### 3.2.2. XRD

**Figure 7:**
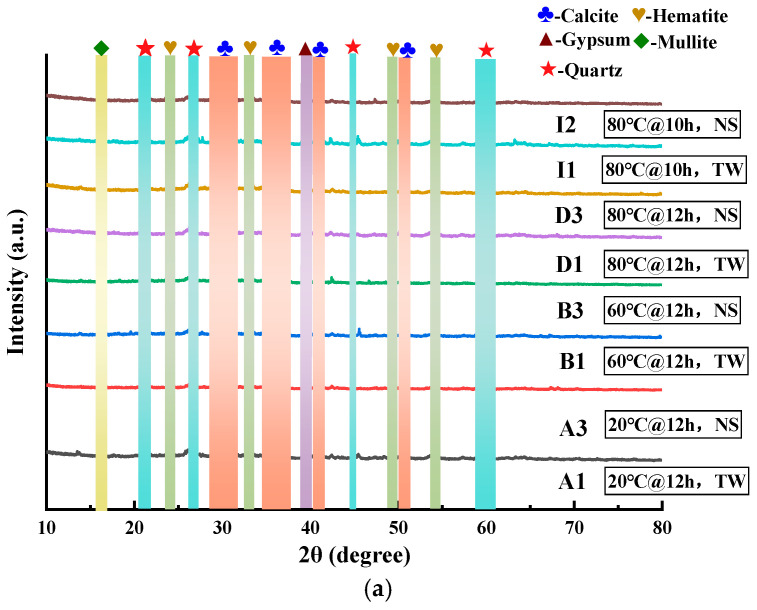

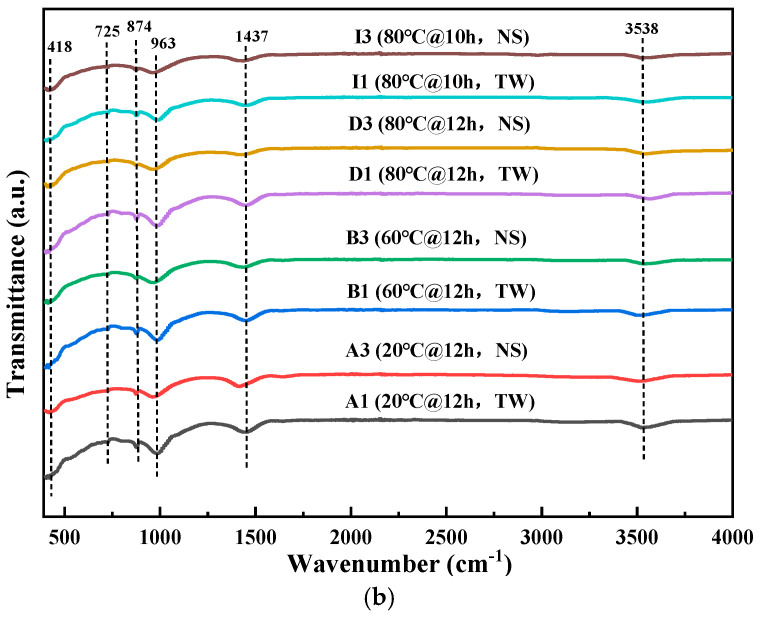
The XRD diffraction spectra (**a**) and FTIR spectral patterns (**b**) of RM-LWA.

The XRD test findings for eight sets of samples (A1, A3, B1, B3, D1, D3, I1, and I3) are shown in Figure 7a. The experimental findings indicate that the typical mineral phases existing within the samples are calcite (CaCO_3_), hematite (Fe_2_O_3_), gypsum (CaSO_4_·2H_2_O), mullite (Al_2_O_3_-SiO_2_), and quartz (SiO_2_).

As depicted in Figure 7a, despite the fact that each sample exhibits the same set of mineral phases internally, the intensities of their characteristic peaks vary significantly. This observation suggests that different curing procedures have no impact on the categories of mineral phases. Instead, they exert an influence on the quantities of these mineral phases, a finding that is in accordance with the previous research results [54,55]. It is observable that, under typical curing settings, the intensity of the calcite diffraction peak in sample A3 is markedly greater in comparison to that in sample A1. Moreover, as the temperature rises, the intensity of the calcite diffraction peak progressively diminishes. This can be explained by the notion that, under high-temperature circumstances, a part of the calcite engages in a relatively vigorous reaction and is converted into calcium compounds. As a consequence, the intensity of its XRD peak is significantly attenuated. The hematite within the RM demonstrated no notable alterations across varying temperatures. The unreacted hematite functioned as a skeletal structure within the ceramsite. In conjunction with the gel products, it contributed to the formation of a more durable and robust microstructure. This finding aligns precisely with the findings of prior studies conducted on RM [56,57]. Furthermore, the C-(A)-S-H and N-A-S-H gels are amorphous in nature. As a result, it proves rather difficult for XRD to detect these non-crystalline hydration products.

#### 3.2.3. FTIR

The FTIR spectral findings for the eight groups of samples (A1, A3, B1, B3, D1, D3, I1, and I3) are presented in Figure 7b. The peak emerging at 3538 cm^−1^ was assigned to the asymmetric stretching vibration of the O-H bond. This phenomenon suggests the existence of interlayer water within the hydroxyl groups [58,59]. Moreover, the symmetric vibrational mode of the C-O bond was responsible for the peak at 1437 cm^−1^ [42,60]. The peak emerging at 874 cm^−1^ was associated with the stretching vibration of the Ti-O bond within TiO_2_ [12]. Moreover, the absorption peaks which are detected at 963, 725, and 418 cm^−1^ are regarded as the contraction vibrations of the Si (Al)-O groups. This has a tight correlation with the amounts of Si and Al situated in the structure [61,62]. The XRD analysis results concur with these findings.

#### 3.2.4. Formation Mechanism of RM-LWA Pore Structure

**Figure 8:**
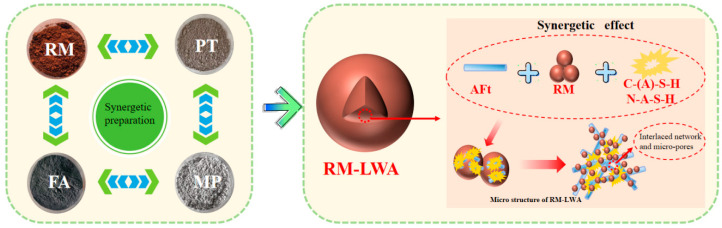
Schematics of the formation of RM-LWA.

The pore structure formation mechanism of RM-LWA is depicted in Figure 8. At the microscopic structural level, the framework of RM-LWA is principally constructed with calcium aluminate hydrates (AFt) acting as the skeletal component, with its component sources clarified by the chemical composition of raw materials: aluminum elements are mainly derived from Al_2_O_3_ (16.53%) in RM; calcium elements originate from CaO in MP (44.05%) and PT (30.35%); sulfate radicals come from SO_3_ (8.81%) in RM, PT, FA, and MP. These three components react to form AFt under the synergistic effect of alkaline components in RM itself (4.46% Na_2_O, 0.11% K_2_O, etc.) and mixed water NS. Meanwhile, industrial solid waste RM acts as a filler to participate in skeleton construction through particle accumulation, and its components such as 50.10% Fe_2_O_3_ also provide support for the skeleton. Industrial solid wastes like RM, on the other hand, function as fillers, jointly constituting the framework structure. The microscopic pores of the aggregate mainly include the pores formed by the accumulation, stacking, and interlaced arrangement within and between the RM aggregates. Alkaline components in RM (such as Na_2_O, K_2_O, etc.) can promote the hydration reaction of materials like MP (containing 44.05% CaO) and enhance their reactivity. Alkaline mixed water NS further promotes the hydration of RM-LWA, significantly reducing the number of unhydrated particles and excess pores in artificial lightweight aggregates [63]. In the process of raw material dissolution, oligomers migrate from the surfaces of RM (containing 14.11% SiO_2_ and 16.53% Al_2_O_3_) and FA (containing 37.16% SiO_2_ and 21.10% Al_2_O_3_) to the pore gaps, followed by repolymerization to form C-S-H gels. During this process, part of the silicon elements in the C-S-H gels is substituted by aluminum elements, leading to the formation of C-A-S-H gels [52]. Specifically, silicon elements are mainly derived from the high SiO_2_ contents in FA (37.16%) and PT (35.12%); aluminum elements come from Al_2_O_3_ in RM and FA; calcium elements are sourced from CaO in MP and PT. The crucial reaction equations are presented in Equations (8)–(10). Under the influence of van der Waals forces, the early-formed gel substances attach to the exterior of unhydrated granules, serving to bind the dispersed raw material particles into a coherent whole. During the stable progression of the hydration reaction, the gel structure solidifies incrementally and transforms into a network structure. Consequently, this process improves the compactness and strength of the artificial lightweight aggregate [64]. Due to the mixed water NS, Na elements displace Ca elements and incorporate into the C-A-S-H gel. This process generates a 3D network N-A-S-H gel having a greater polymerization degree, and it maximally fills the gaps between RM particles, rendering the internal structure more compact. Ultimately, this phenomenon contributes to the enhancement of the RM-LWA’s strength [53]. The key reaction equation is presented in Equation (11).

(8) $$\mathrm{CaO}+{\mathrm{SiO}}_{2}\to {\mathrm{Ca}}_{3}{\mathrm{SiO}}_{5}$$

(9) $$Ca_{3}SiO_{5}+H_{2}O\to C-S-H\;gel$$

(10) $$Ca_{3}SiO_{5}+Al_{2}O_{3}+H_{2}O\to C-A-S-H\;\mathrm{gel}$$

(11) $$Ca_{3}SiO_{5}+H_{2}O+NaOH\to N-A-S-H\;gel$$

### 3.3. Freeze–Thaw Resistance

**Figure 9:**
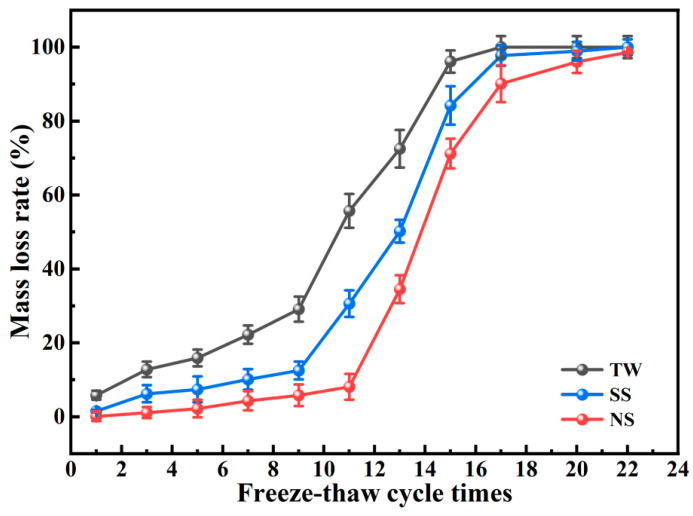
Freeze–thaw resistance of RM-LWA.

In the context of standard curing conditions, the mass reductions of the three kinds of RM-LWA after undergoing freeze–thaw cycles are depicted in Figure 9. Upon undergoing 20 freeze–thaw cycles, RM-LWA fabricated by adding mixed water SS experiences a mass loss of 100%. This clearly shows that the frost resistance of this particular aggregate is rather weak. In comparison, RM-LWA produced with the addition of mixed water NS showcases enhanced frost resistance. Only after 22 freeze–thaw cycles does its mass loss reach 100%. This phenomenon is chiefly associated with the macropores and microfractures present on the aggregate’s surface. Obviously, the microcracks on the surface and the internal micropores of the aggregate are the main channels for water absorption. In a low-temperature environment, the water-saturated aggregate will generate frost heaving force due to freezing and expansion. Excessive frost heaving force may cause the aggregate to crack, further damaging the pore walls and forming microcracks [65]. As the freeze–thaw cycles progress, these microcracks gradually increase in number and begin to expand. Ultimately, the air pores become interconnected, giving rise to macroscopic cracks. This phenomenon results in the peeling off of the aggregate surface and a degradation of its mechanical properties [66]. In addition, the presence of internal pores in RM-LWA can also play a certain positive role. Specifically, the internal pores of RM-LWA can reduce the compactness of the aggregate and decrease its water saturation. Furthermore, by alleviating frost heave stress, they mitigate the damage suffered by RM-LWA during freeze–thaw cycles, ultimately effectively improving the freeze–thaw resistance of RM-LWA [67].

### 3.4. Leaching Properties of Heavy Metals

**Figure 10:**
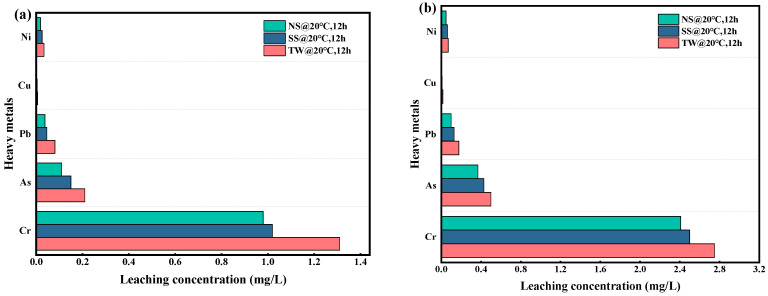
The heavy metal leaching levels under non-freeze–thaw cycling (**a**) and freeze–thaw cycling (**b**) conditions.

Heavy metal leaching toxicity experiments were carried out through the ICP-OES for evaluating the effects of RM-LWA on environmental safety. Figure 10 shows the outcomes of heavy metal leaching tests conducted on RM-LWA cured under conditions without freeze–thaw cycling and with freeze–thaw cycling. In compliance with the Chinese Identification Standards for Hazardous Wastes (GB 5085.3-2007) [68], the leaching levels of Cu (<100 mg/L), Cr (<15 mg/L), As (<5 mg/L), Ni (<5 mg/L), and Pb (<5 mg/L) in the three varieties of RM-LWA all meet the limits mentioned in the standard for the discharge of non-hazardous substances into the environment.

As shown in Figure 10, under non-freeze–thaw cycle conditions, the leaching concentrations of Cr in samples A1 (TW@20 °C, 12 h), A2 (SS@20 °C, 12 h), and A3 (NS@20 °C, 12 h) were the highest, reaching 1.31 mg/L, 1.02 mg/L, and 0.98 mg/L, respectively, while the leaching concentration of heavy metal Cu was the lowest. With freeze–thaw cycles, the heavy metal Cr kept the highest leaching level, and the leaching amounts of Cr and heavy metals As, Pb, Cu, and Ni all showed a significant increasing trend. It is worth noting that, whether under freeze–thaw cycle or non-freeze–thaw cycle conditions, the heavy metal leaching concentration of RM-LWA produced with mixed water NS stayed consistently lower than those of other samples. This phenomenon could be ascribed to the alkaline nature of the mixed aqueous NS, which intensified the reaction kinetics of active components and facilitated the formation of additional gel-like products [69,70] and made the internal structure of RM-LWA denser, and thus it was better able to enclose heavy metals and lower the risk of their leaching.

## 4. Risks, Mitigation Strategies, and Application FIELDS of RM-LWA

### 4.1. Risks, Mitigation Strategies

Although the heavy metal leaching concentration of RM-LWA prepared in this study is far lower than the limit specified in the current standard GB 5085.3-2007, to further mitigate the risk of heavy metal migration during long-term service, it is proposed to apply interface encapsulation treatment on RM-LWA particles using a functional coating [71,72,73]. After curing, this coating forms a high-density and low-permeability barrier around the RM-LWA particles, which can reduce the amount of heavy metal leaching and significantly enhance the long-term environmental stability of the material. Meanwhile, applying a water-based epoxy coating on RM-LWA-based products can reduce water absorption by forming a hydrophobic layer, thereby improving the frost resistance of the material.

### 4.2. Application Fields

#### 4.2.1. Non-Structural Fill Materials

RM-LWA exhibits a low bulk density and moderate compressive strength, making it suitable for non-structural fill applications such as backfilling in road embankments, pipeline trenches, or building foundations. Its lightweight nature reduces lateral pressure on surrounding structures, while its porous structure can help mitigate water accumulation through drainage, enhancing the stability of fill layers.

#### 4.2.2. Insulation Blocks and Wall Panels

The porous microstructure of RM-LWA contributes to low thermal conductivity, which is favorable for thermal insulation. It can be incorporated into lightweight concrete to produce insulation blocks or prefabricated wall panels for residential and commercial buildings. These components can improve indoor thermal comfort and reduce energy consumption for heating or cooling, aligning with green building standards.

#### 4.2.3. Eco-Friendly Backfill in Landscaping and Civil Engineering

As RM-LWA is fabricated from industrial solid wastes (RM, FA, etc.), its use in landscaping backfill (e.g., around plants, in green roofs) or civil engineering filler materials promotes waste recycling and reduces environmental impact. Its porous structure also supports water retention, benefiting vegetation growth in landscaping applications.

## 5. Conclusions

In this research, RM, PT, FA, and MP were employed as raw materials to fabricate non-fired RM-LWA. A comprehensive investigation was conducted into their mineral composition, physical properties, microstructural features, freeze–thaw durability, and heavy metal leachability. The results of this research offer a scientific foundation and theoretical direction for the collaborative use of solid wastes like RM. The following conclusions are reached:

(1) As opposed to TW and SS, when the curing conditions were standard, the cylinder crush strength value for RM-LWA samples made by adding NS increased by 33% and 14%, respectively. In addition, when steam-cured at 80 °C, the cylinder crush strength of lightweight aggregates obtained by incorporating NS exceeded 90% of the value at a 90 °C curing temperature, indicating that further increasing the temperature has a limited impact on the strength. At this time, the softening coefficient is 0.94, and the 1 h water absorption rate reaches 14%.
(2) Microscopic tests showed that the maintenance method did not change the type of mineral phases produced but changed their quantity. The hydrating substances identified in RM-LWA primarily comprise N-A-S-H and C-(A)-S-H. During the hydration process, a robust colloidal framework is generated and uniformly distributed across the particle surfaces, thereby improving the strength to a certain degree.
(3) With standard curing conditions, RM-LWA containing NS exhibited superior freeze–thaw resistance, showing a complete 100% mass loss following 22 freeze–thaw cycles. Heavy metal leachability assays reveal that the elution concentrations of heavy metals (Cr, Pb, As, Cu, and Ni) in RM-LWA specimens all conform to the regulatory thresholds for non-hazardous waste disposal into the environment.

## 6. Future Perspectives

This study has achieved certain results in the preparation and performance exploration of RM-LWA. However, there are still some directions that can be further expanded and studied in depth, providing broad prospects for future research:

(1) Building on the existing findings of this study, future research will focus on the neglected alkali metals (such as K, Na) in RM, conducting in-depth investigations into their impacts on the environment.
(2) RM, PT, FA, and MP have been studied for RM-LWA, but exploring more solid waste types can broaden sources and improve resource efficiency. Further research on material proportions, characteristics, and interactions is needed for higher-performance RM-LWA.
(3) Steam-cured artificial lightweight aggregates have high early strength (shortening construction periods) but limited late-stage strength. Future research should explore modified coatings to improve performance.
(4) Future research will conduct 6–8-month RM-LWA field exposure tests under simulated diverse climates to monitor heavy metal leaching under natural stressors.

## Figures and Tables

**Table 1:** The chemical composition of RM, PT, FA, and MP.

Material/wt%	CaO	SiO_2_	Al_2_O_3_	Fe_2_O_3_	SO_3_	MgO	TiO_2_	K_2_O	Na_2_O	LOI^a^
RM	0.48	14.11	16.53	50.10	0.58	0.13	4.84	0.11	4.46	9.16
PT	30.35	35.12	13.32	1.42	1.36	7.94	1.58	1.84	0.34	7.26
FA	20.88	37.16	21.10	3.58	3.36	3.87	2.75	4.08	2.15	1.43
MP	44.05	21.95	16.68	0.15	3.51	9.30	1.05	0.28	0.79	-

LOI^a^ = loss on ignition.

**Table 2:** Mixing proportions and curing regimes of RM-LWA.

RM-LWA	Type of Additive	Curing Condition
A1	TW	Standard curing at 20 °C over a 12 h period
A2	SS	
A3	NS	
B1	TW	Curing with steam at 60 °C over a 12 h period
B2	SS	
B3	NS	
C1	TW	Curing with steam at 70 °C over a 12 h period
C2	SS	
C3	NS	
D1	TW	Curing with steam at 80 °C over a 12 h period
D2	SS	
D3	NS	
E1	TW	Curing with steam at 90 °C over a 12 h period
E2	SS	
E3	NS	
F1	TW	Curing with steam at 80 °C over a 4 h period
F2	SS	
F3	NS	
G1	TW	Curing with steam at 80 °C over a 6 h period
G2	SS	
G3	NS	
H1	TW	Curing with steam at 80 °C over an 8 h period
H2	SS	
H3	NS	
I1	TW	Curing with steam at 80 °C over a 10 h period
I2	SS	
I3	NS	

**Table 3:** An undertaking of physical property comparison between the present work and other relevant literature.

Raw Materials	Sintering Temperature (°C)	The *ρ*_ap_ Value (kg/m^3^)	1 h Water Absorption (%)	Crush Strength (MPa)	References
Silica fume	1120	691	1.33	5.54	[46]
Lime mud	1050	740	39.03	4.73	[44]
Waste glass	1125	482	7.2	1.43	[47]
Nepheline tailings	1150	410	3.5	1.8	[48]
Bayan Obo tailings	1130	1940	31.42	1.89	[49]
Red mud	1150	450	9.0	1.12	[50]
Engineering excavated soil	1175	967.1	11.39	5.02	[45]

## Data Availability

The original contributions presented in this study are included in the article. Further inquiries can be directed to the corresponding authors.

## References

[1] Lima M.S., Thives L.P. (2020). Evaluation of red mud as filler in Brazilian dense graded asphalt mixtures. Constr. Build. Mater..

[2] Vigneshwaran S., Uthayakumar M., Arumugaprabu V. (2019). Development and sustainability of industrial waste-based red mud hybrid composites. J. Clean. Prod..

[3] Kim C.-J., Chung C.-S., Jung J.-M., Kim Y.-R., Kang D.-W., Kim H.-E., Shin K.-H., Choi K.-Y. (2023). Long-term effects of chromium from red mud (bauxite residue) ocean dumping on the benthic environment in South Korea. Mar. Pollut. Bull..

[4] Shi Y., Guo W., Jia Y., Xue C., Qiu Y., Zhao Q., Wang D. (2022). Preparation of non-sintered lightweight aggregate ceramsite based on red mud-carbide slag-fly ash: Strength and curing method optimization. J. Clean. Prod..

[5] Liu X., Zhang N. (2011). Utilization of red mud in cement production: A review. Waste Manag. Res..

[6] Jia K., Zhou Z., Singh S.V., Wang C. (2024). A review of the engineered treatment of red mud: Construction materials, metal recovery, and soilization revegetation. Results Eng..

[7] Pan X., Wu H., Lv Z., Yu H., Tu G. (2023). Recovery of valuable metals from red mud: A comprehensive review. Sci. Total Environ..

[8] Padhan A., Paul B. (2025). Unlocking the potential of red mud: Advanced strategies for economic optimization and sustainable recovery of critical minerals. J. Environ. Manag..

[9] Swain B., Akcil A., Lee J.-C. (2022). Red mud valorization an industrial waste circular economy challenge; review over processes and their chemistry. Crit. Rev. Environ. Sci. Technol..

[10] Bose B.P. (2024). Comprehensive Utilizations of Red Mud with Emphasis on Circular Economy: An Approach towards Achieving the United Nations Sustainable Development Goals. Int. J. Earth Sci. Knowl. Appl..

[11] Swain B. (2022). Red mud: An environmental challenge but overlooked treasure for critical rare earth metals. MRS Bull..

[12] Yang J., Wang Z., Luo H., Wang H., Chen L., Liu M., Tang M., He B.-J. (2024). Preparation and properties of alkali-activated red mud-based artificial lightweight aggregates. Constr. Build. Mater..

[13] Ma M., Tam V.W., Le K.N., Li W. (2020). Challenges in current construction and demolition waste recycling: A China study. Waste Manag..

[14] Aslam M.S., Huang B., Cui L. (2020). Review of construction and demolition waste management in China and USA. J. Environ. Manag..

[15] Zhang J., Yao Z., Wang K., Wang F., Jiang H., Liang M., Wei J., Airey G. (2021). Sustainable utilization of bauxite residue (Red Mud) as a road material in pavements: A critical review. Constr. Build. Mater..

[16] Xie W., Zhou F., Liu J., Bi X., Huang Z., Li Y., Chen D., Zou H., Sun S. (2020). Synergistic reutilization of red mud and spent pot lining for recovering valuable components and stabilizing harmful element. J. Clean. Prod..

[17] Khairul M., Zanganeh J., Moghtaderi B.M. (2019). The composition, recycling and utilisation of Bayer red mud. Resour. Conserv. Recycl..

[18] Wang L., Sun N., Tang H., Sun W. (2019). A review on comprehensive utilization of red mud and prospect analysis. Minerals.

[19] Molineux C.J., Newport D.J., Ayati B., Wang C., Connop S.P., Green J.E. (2016). Bauxite residue (red mud) as a pulverised fuel ash substitute in the manufacture of lightweight aggregate. J. Clean. Prod..

[20] Sun Y., Li J.-S., Chen Z., Xue Q., Sun Q., Zhou Y., Chen X., Liu L., Poon C.S. (2021). Production of lightweight aggregate ceramsite from red mud and municipal solid waste incineration bottom ash: Mechanism and optimization. Constr. Build. Mater..

[21] Liu S., Yang C., Liu W., Yi L., Qin W. (2020). A novel approach to preparing ultra-lightweight ceramsite with a large amount of fly ash. Front. Environ. Sci. Eng..

[22] Zhao Q., Shi Y., Xue C., Jia Y., Guo W., Wang D., Wang S., Gao Y. (2023). Investigation of various curing methods on the properties of red mud-calcium carbide slag-based artificial lightweight aggregate ceramsite fabricated through alkali-activated cold-bonded pelletization technology. Constr. Build. Mater..

[23] Gesoğlu M., Güneyisi E., Mahmood S.F., Öz H.Ö., Mermerdaş K. (2012). Recycling ground granulated blast furnace slag as cold bonded artificial aggregate partially used in self-compacting concrete. J. Hazard. Mater..

[24] Zhang X., Zhang C., Hu Z., Wang Z., Cao Z., Wang W. (2025). Production of a low-carbon and economical high-strength artificial aggregate from gold tailings for the preparation of lightweight aggregate concrete. Cem. Concr. Compos..

[25] Deng X., Li J., Du D., Wang T. (2024). Manufacturing non-sintered ceramsite from dredged sediment, steel slag, and fly ash for lightweight aggregate: Production and characterization. Environ. Sci. Pollut. Res. Int..

[26] Shenwei Z., Linying Y., Haibin H., Yiping Z., Lei H., Yujia Z., Yajing Y., Jianli J. (2019). Preparation and environmental toxicity of non-sintered ceramsite using coal gasification coarse slag. Arch. Environ. Prot..

[27] Małgorzata F., Danuta B.-H., Magdalena W. (2016). Utilization of sewage sludge in the manufacture of lightweight aggregate. Environ. Monit. Assess..

[28] Luisa B., Alessandro B., Fernanda A., Isabella L., Grazia G., Maria T.C.P., Carmen M.G. (2020). Environmental impact estimation of ceramic lightweight aggregates production starting from residues. Int. J. Appl. Ceram. Technol..

[29] Kalle K., Abhijit M., Mirja I., Priyadharshini P. (2024). Utilization of fine concrete waste as a lightweight aggregate via granulation: Technical and environmental assessment. J. Clean. Prod..

[30] Tesovnik A., Ottosen L.M., Ducman V. (2025). Carbonation of lightweight alkali-activated aggregates based on biomass fly ash: Effect on microstructure and leaching behavior. Case Stud. Constr. Mater..

[31] (2010). Lightweight Aggregates and Its Test Methods—Part 1: Lightweight Aggregates.

[32] (2010). National Standards of the People’s Republic of China, Lightweight Aggregates and Its Test Methods—Part 2. Test Methods for Lightweight Aggregate.

[33] (2009). Standard for Test Methods of Long-Term Performance and Durability of Ordinary Concrete.

[34] (2010). Solid Waste-Extraction Procedure for Leaching Toxicity-Horizontal Vibration Method.

[35] Huang W., Wang H. (2021). Geopolymer pervious concrete modified with granulated blast furnace slag: Microscale characterization and mechanical strength. J. Clean. Prod..

[36] Rehman M.U., Rashid K., Haq E.U., Hussain M., Shehzad N. (2020). Physico-mechanical performance and durability of artificial lightweight aggregates synthesized by cementing and geopolymerization. Constr. Build. Mater..

[37] Chuan H.D.L., Abd R.R., Marwan K., Zarina Y., Bakri A.M.M.A., Doru B.N.D., Hamzah F., Ratna E., Rosnita M., Alida A. (2022). Artificial Lightweight Aggregates Made from Pozzolanic Material: A Review on the Method, Physical and Mechanical Properties, Thermal and Microstructure. Materials.

[38] Abbas W., Khalil W., Nasser I. (2018). Production of lightweight Geopolymer concrete using artificial local lightweight aggregate. MATEC Web Conf..

[39] Risdanareni P., Schollbach K., Wang J., Belie N.D. (2020). The effect of NaOH concentration on the mechanical and physical properties of alkali activated fly ash-based artificial lightweight aggregate. Constr. Build. Mater..

[40] Bekkeri G.B., Shetty K.K., Nayak G. (2023). Synthesis of artificial aggregates and their impact on performance of concrete: A review. J. Mater. Cycles Waste Manag..

[41] Hu S., Gong X., Li Q., Fan Z. (2024). Preparation and Characterization of Non-Sintered Ceramsites From Alkali-Activated Foundry Dust. Int. J. Met..

[42] Liu J., Niu R., Hu J., Ren Y., Zhang W., Liu G., Li Z., Xing F., Ren J. (2023). The performance and microstructure of alkali-activated artificial aggregates prepared from municipal solid waste incineration bottom ash. Constr. Build. Mater..

[43] Ying K.S., Hanizam A. (2021). Utilisation of Recycled Silt from Water Treatment and Palm Oil Fuel Ash as Geopolymer Artificial Lightweight Aggregate. Sustainability.

[44] Qin J., Cui C., Cui X., Hussain A., Yang C. (2015). Preparation and characterization of ceramsite from lime mud and coal fly ash. Constr. Build. Mater..

[45] Jin J., Ying W., Jiawei L., Ming W., Chuanlin W., Wei B. (2024). Characterization and mechanism of sintered light aggregate ceramsite with engineering excavated soil. Structures.

[46] Zhuo L., Rongxin G., Xiao-Yong W., Chaoshu F., Run-Sheng L. (2023). Construction ceramsite from low-silicon red mud: Design, preparation, and sintering mechanism analysis. Process Saf. Environ. Prot..

[47] Pei J., Ye P., Lu B., Lu W., Jiang F., Lu Z., Pan X., Xu Z. (2025). Ultra-lightweight ceramsites from waste glass and red mud: Performance and microstructural analysis. J. Environ. Chem. Eng..

[48] Jun J., Song C., Chao J., Gui W., Tie L., Tianyuan X., Luo L., Wenjing D., Xu Y., Tao D. (2023). Preparation and properties of high-strength lightweight aggregate ceramsite from nepheline tailings. Constr. Build. Mater..

[49] Fan C.Y., Xian H.W., Hao Z.Y., Ci W.Y., Jun P., Li A.S. (2023). Process and property optimization of ceramsite preparation by Bayan Obo tailings and blast furnace slag. J. Iron Steel Res. Int..

[50] Jiannan P., Xiaolin P., Yibo W., Zhongyang L., Haiyan Y., Ganfeng T. (2023). Effects of alkali and alkaline-earth oxides on preparation of red mud based ultra-lightweight ceramsite. Ceram. Int..

[51] Liu B., Zhang Q., Feng Y., Chen Q., Guo L. (2024). Mechanical and microstructural analysis of cemented tailings backfill by copper slag through alkaline activation emphasizing red mud. Constr. Build. Mater..

[52] Xu D., Ni W., Wang Q., Xu C., Li K. (2021). Ammonia-soda residue and metallurgical slags from iron and steel industries as cementitious materials for clinker-free concretes. J. Clean. Prod..

[53] Walkley B., Nicolas R.S., Sani M.-A., Rees G.J., Hanna J.V., van Deventer J.S., Provis J.L. (2016). Phase evolution of C-(N)-ASH/NASH gel blends investigated via alkali-activation of synthetic calcium aluminosilicate precursors. Cem. Concr. Res..

[54] Guo W., Wang S., Xu Z., Zhang Z., Zhang C., Bai Y., Zhao Q. (2021). Mechanical performance and microstructure improvement of soda residue–carbide slag–ground granulated blast furnace slag binder by optimizing its preparation process and curing method. Constr. Build. Mater..

[55] Abdelfattah M.M., Géber R., Abdel-Kader N.A., Kocserha I. (2022). Assessment of the mineral phase and properties of clay-Ca bentonite lightweight aggregates. Arab. J. Geosci..

[56] Nie Q., Hu W., Ai T., Huang B., Shu X., He Q. (2016). Strength properties of geopolymers derived from original and desulfurized red mud cured at ambient temperature. Constr. Build. Mater..

[57] Sglavo V.M., Campostrini R., Maurina S., Carturan G., Monagheddu M., Budroni G., Cocco G. (2000). Bauxite ‘red mud’in the ceramic industry. Part 1: Thermal behaviour. J. Eur. Ceram. Soc..

[58] Wang J., Liu E., Li L. (2018). Multiscale investigations on hydration mechanisms in seawater OPC paste. Constr. Build. Mater..

[59] Zhai Q., Kurumisawa K. (2021). Effect of accelerators on Ca(OH)_2_ activated ground granulated blast-furnace slag at low curing temperature. Cem. Concr. Compos..

[60] Choudhary H.K., Anupama A., Kumar R., Panzi M., Matteppanavar S., Sherikar B.N., Sahoo B. (2015). Observation of phase transformations in cement during hydration. Constr. Build. Mater..

[61] Kim H.-S., Kim K.-S., Jung S.S., Hwang J.I., Choi J.-S., Sohn I. (2015). Valorization of electric arc furnace primary steelmaking slags for cement applications. Waste Manag..

[62] Guo W., Zhang Z., Bai Y., Zhao G., Sang Z., Zhao Q. (2021). Development and characterization of a new multi-strength level binder system using soda residue-carbide slag as composite activator. Constr. Build. Mater..

[63] Zhong M., Meng J., Ning B., Na F., Cui T., Shi X., Cui T. (2024). Preparation and alkali excitation mechanism of coal gangue-iron ore tailings non-sintering ceramsite. Constr. Build. Mater..

[64] Zhang N., Li H., Liu X. (2016). Hydration mechanism and leaching behavior of bauxite-calcination-method red mud-coal gangue based cementitious materials. J. Hazard. Mater..

[65] Tian Y., Qin Z., Lin Z., Shen P., Chen L., Chen G., Zhang L., Gao J., Liu S., Yang N. (2024). Study on the physical mechanical properties and freeze-thaw resistance of energy storage concrete with artificial phase change aggregate. J. Build. Eng..

[66] Tian Y., Lai Y., Pei W., Qin Z., Li H. (2022). Study on the physical mechanical properties and freeze-thaw resistance of artificial phase change aggregates. Constr. Build. Mater..

[67] Yeon J.H., Kim K.-K. (2018). Potential applications of phase change materials to mitigate freeze-thaw deteriorations in concrete pavement. Constr. Build. Mater..

[68] (2007). Identification Standards for Hazardous Wastes-Identification for Extraction Toxicity.

[69] Ji H., Zhang Y., Ren R., Guo Y., Du Y., Zhao X., Feng G. (2025). Preparation and performance influencing factors of multi-component solid waste based non-sintered ceramsites. Constr. Build. Mater..

[70] Liu J., Gao Y., Wang Y., Zhao J. (2024). Non-sintered ceramsite from alkali-activated gasification slag for adsorption through the regulation of physical properties and pore structure. J. Clean. Prod..

[71] Feng C., Wang J., Chen M., Zhao X., Zhang W., Zhu J., Du M. (2025). Study on the modification effect of recycled fine aggregates treated by synergistic alkali activation using volcanic ash and cement powder wrapping. J. Mater. Res. Technol..

[72] Elias L., Bijimol B.I., Geethanjali C.V., Anil A., Bhagya T.C., Shibli S.M.A. (2025). Fine-recycled concrete aggregate based cementitious polymer composite coating for corrosion and biocorrosion protection of concrete reinforcing steel bar. Constr. Build. Mater..

[73] Colangelo F., Messina F., Cioffi R. (2015). Recycling of MSWI fly ash by means of cementitious double step cold bonding pelletization: Technological assessment for the production of lightweight artificial aggregates. J. Hazard. Mater..

